# Osteochondroma of the distal tibia in an 8‐year‐old child: Do we need to excise a benign tumor?

**DOI:** 10.1002/ccr3.3321

**Published:** 2020-09-09

**Authors:** Nikiforos Galanis, Ioannis Delniotis, George Noussios, Anastasios Katsourakis, Dimitrios Kitridis, Benedikt Leidinger, Panagiotis Givissis

**Affiliations:** ^1^ Department of Orthopaedics George Papanikolaou Hospital Aristotle University of Thessaloniki Thessaloniki Greece; ^2^ Department of Paediatric Orthopaedics, Neuro‐Orthopaedics, Foot & Ankle Surgery Orthopaedic Hospital Volmarstein Wetter Germany; ^3^ School of Physical Education and Sports Sciences of Serres Aristotle University of Thessaloniki Thessaloniki Greece; ^4^ Department of Surgery Agios Dimitrios General Hospital Thessaloniki Greece

**Keywords:** child, osteochondroma, surgical excision, tumor

## Abstract

Osteochondromas are benign tumors that can be responsible for angular deformities, limb‐length discrepancy, and impending fractures of the neighboring bones. The risk of future fractures and joint malalignment is an indication for surgical intervention.

## Graphical abstract

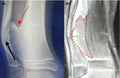

## 1 CLINICAL IMAGE

An 8‐year‐old boy (no medical history) presented with a painless swelling of his right ankle without any history of trauma. On the posterolateral aspect of his distal tibia was a swelling noticed with an obvious bowing deformity of his fibula. The function and the neuromuscular status of his ankle and foot were normal.

**FIGURE 1:**
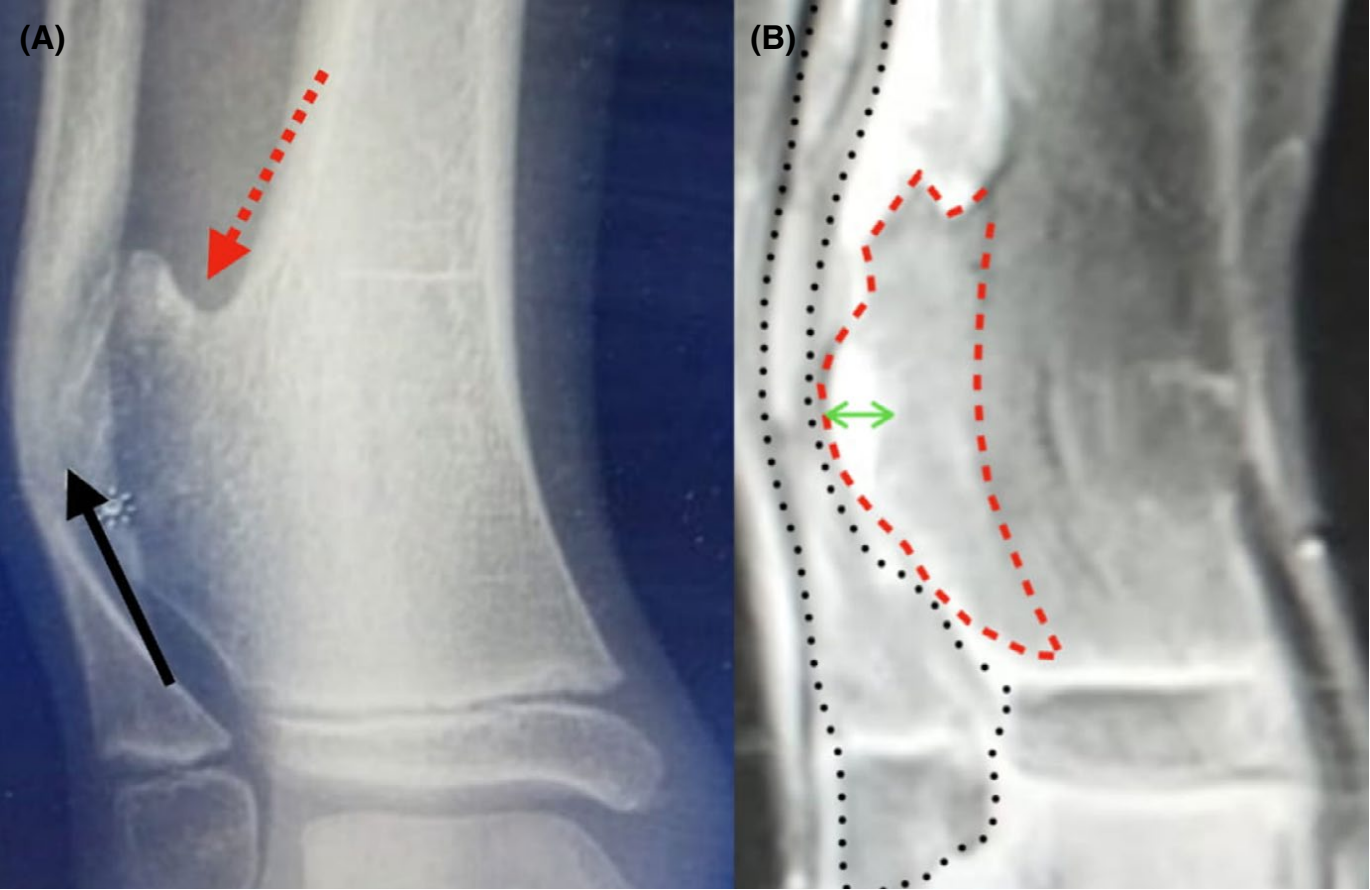
A, AP view of the distal tibia. The red dotted arrow shows the osteochondroma of our patient. The black arrow shows a significant bowing and weakening of the fibula. B, MRI view of the distal tibia of our patient. The red dotted circle shows the osteochondroma that expands and “pressures” the fibula. The green arrow shows the cartilage cap, which should always be measured. The black dots indicate the bowing of the fibula

**FIGURE 2:**
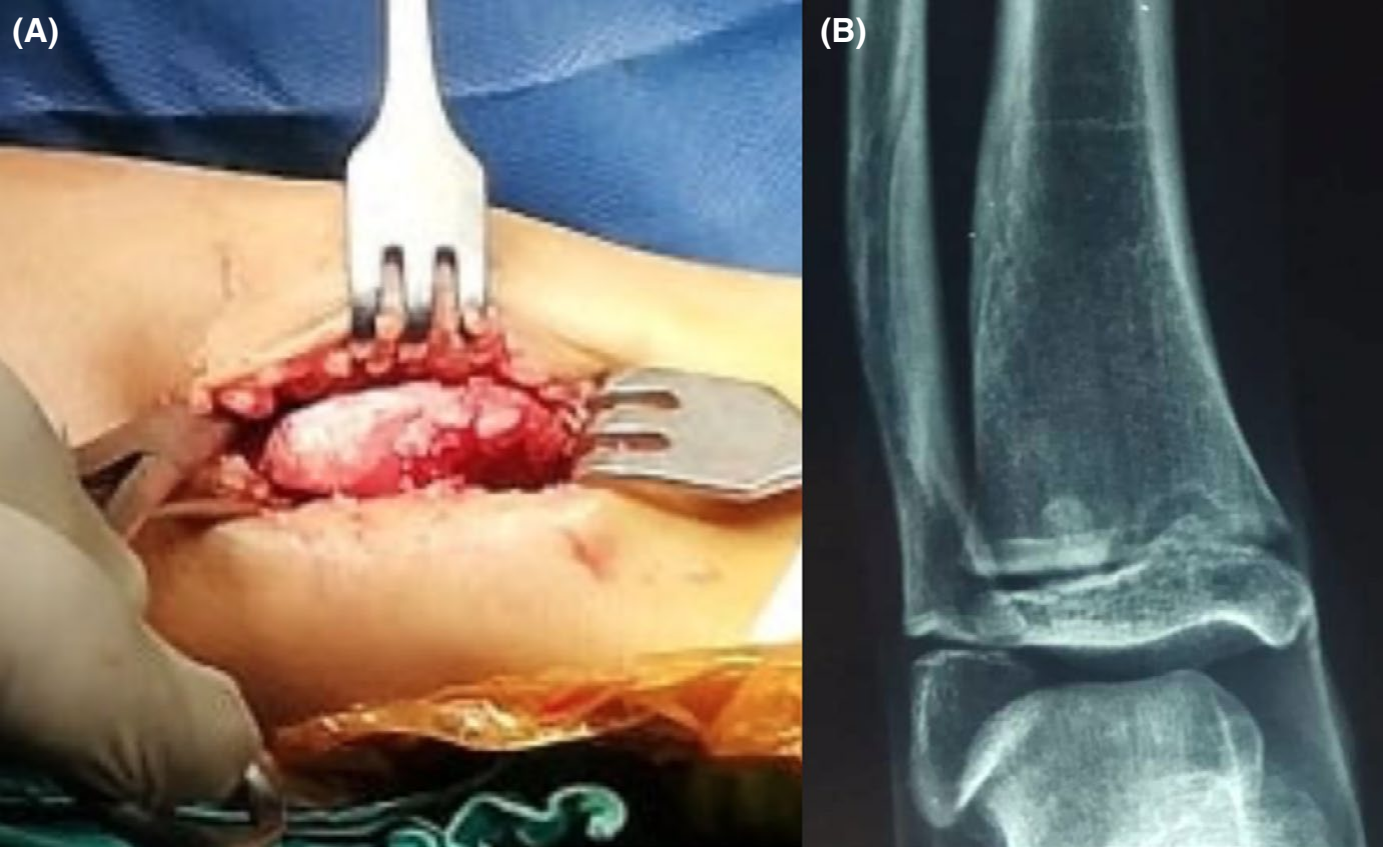
A, Intra‐operative image, showing, under direct vision, the expanding osteochondroma of the distal tibia. B, Postoperative X‐ray image of the distal tibia at 6‐month follow‐up. The fibula has already started to remodel to its normal anatomy

X‐rays of the distal tibia revealed an osteochondroma and a significant erosion of the distal fibula (Figure 1A). Full‐limb radiographs did not show any tumors. So, a solitary osteochondroma of the right distal fibula was diagnosed. MRI was also performed to assess the cartilage thickness (5‐6 mm) (Figure 1B). Surgical treatment was chosen (tibia posterolateral approach) due to the impending fracture of the fibula (Figure 2A). At 6‐month follow‐up, the fibula has already started to remodel to its original normal shape (Figure 2B).

Solitary osteochondromas are one of the most common osseous tumors accounting for up to 30%. They are usually asymptomatic and require only regular follow‐up. Indications for surgical excision are as follows: a) large and symptomatic (pain) osteochondromas, b) osteochondromas that are responsible for limb‐length discrepancy and/or angular deformities (genu varum/valgum), c) growing osteochondromas in skeletally mature patients, and d) cartilage caps greater than 1,5‐2 cm, especially in skeletally mature patients, measured by MRI imaging.^1^

Early surgical excision of a distal tibia metaphysis osteochondroma that deforms and weakens the fibula should be performed to prevent future fracture or varus/valgus deformities of the ankle joint.^2^

## CONFLICT OF INTEREST

None declared.

## AUTHOR CONTRIBUTIONS

NG: drafted the manuscript and contributed to patient care. ID: drafted the manuscript. GN, AK, and DK: obtained the photographs and contributed to patient care. BL: revised the manuscript. PG: approved the final version to be published.
